# RETRACTION: Meta‐Analysis on GLP‐1 Mediated Modulation of Autophagy in Islet β‐Cells: Prospectus for Improved Wound Healing in Type 2 Diabetes

**DOI:** 10.1111/iwj.70546

**Published:** 2025-04-18

## Abstract

**RETRACTION:** XiaW. , YuH. , and WenP. , “Meta‐Analysis on GLP‐1 Mediated Modulation of Autophagy in Islet β‐Cells: Prospectus for Improved Wound Healing in Type 2 Diabetes,” International Wound Journal 21, no. 4 (2024): e14841, 10.1111/iwj.14841.38512120 PMC10956537

The above article, published online on 21 March 2024, in Wiley Online Library (http://onlinelibrary.wiley.com/), has been retracted by agreement between the journal Editor in Chief, Professor Keith Harding; and John Wiley & Sons Ltd. Following an investigation by the publisher, all parties have concluded that this article was accepted solely on the basis of a compromised peer review process. The editors have therefore decided to retract the article. The authors did not respond to our notice regarding the retraction.

**RETRACTION:** W. Xia , H. Yu , and P. Wen , “Meta‐Analysis on GLP‐1 Mediated Modulation of Autophagy in Islet β‐Cells: Prospectus for Improved Wound Healing in Type 2 Diabetes,” International Wound Journal 21, no. 4 (2024): e14841, 10.1111/iwj.14841.38512120 PMC10956537

The above article, published online on 21 March 2024, in Wiley Online Library (http://onlinelibrary.wiley.com/), has been retracted by agreement between the journal Editor in Chief, Professor Keith Harding; and John Wiley & Sons Ltd. Following an investigation by the publisher, all parties have concluded that this article was accepted solely on the basis of a compromised peer review process. The editors have therefore decided to retract the article. The authors did not respond to our notice regarding the retraction.

